# Reproducibility of interactive registration of 3D CT and MR pediatric treatment planning head images

**DOI:** 10.1120/jacmp.v2i3.2606

**Published:** 2001-09-01

**Authors:** Jaap Vaarkamp

**Affiliations:** ^1^ Department of Radiation Oncology St. Jude Children's Research Hospital 332 North Lauderdale Street Memphis Tennessee 38105‐2794

**Keywords:** matching, image registration, radiotherapy treatment planning, accuracy

## Abstract

The reproducibility of an interactive image registration technique used as part of the radiotherapy treatment planning process was investigated for 3D CT and MR pediatric head images. Over a nine month period, 85 CT/MR image registrations, required for treatment planning, were repeated, 52 by the same operator and 33 by a different operator. All were performing image registrations for normal clinical care and the first registration was used clinically. Inter‐ and intra‐operator reproducibility of the translation and rotation were calculated separately. The standard deviation of the average total translation and rotation was 0.39 mm and 1.7°, and 0.58 mm and 2.8°, respectively. The maximum difference between registrations was 1.1 mm and 4.1° when repeated by the same operator, and 1.4 mm and 5.8° when repeated by another operator. The variation for the lowest resolution parameters, out of plane translation and rotations, was 2 to 3 times larger than for in‐plane movements. A registration took between 5 minutes and over half an hour for difficult cases, with a mean of 14.3 minutes. One to two millimeter reproducibility was not achieved and interactive registration was relatively time consuming. There is a clear image resolution effect on registration reproducibility, suggesting that reducing slice thickness could considerably improve registration reproducibility.

PACS number(s): 87.53.–j, 87.57. –s

## INTRODUCTION

The combined use of computed tomography (CT) data for dose calculations and magnetic resonance (MR) data for the identification of the target volume is nowadays considered an essential part of radiotherapy treatment planning.^1^ Yet, to use CT and MR images this way, accurate image registration is required because any misalignment at this stage will become a systematic error during the further patient treatment. Most commercial treatment planning systems now include some manual or interactive registration facility^2^ ^–^ ^4^ and in the Radiation Oncology department of St. Jude Children's Research Hospital, an interactive technique is used for image registration. As part of an increased emphasis on quality assurance the reproducibility of the used registration technique needed to be quantified. In the literature, there is some data on point‐based manual techniques,57 on automatic techniques,^8^ ^–^ ^12^ and extensive reviews on image registration techniques in general.^13^ ^,^ ^14^ However, no studies were found on the reproducibility of an interactive approach where CT and MR are presented in one image using a color wash display, nor was a study found that reports MR‐to‐CT registration reproducibility levels for a substantial radiotherapy patient population.

One important drawback of manual techniques is that they are operator time consuming.6 Hence, applying automated techniques, for example based on objective functions, could potentially decrease the overall alignment errors introduced by image registration and, secondly, also reduce the time demand for this frequent task. However, before switching from a manual to an automatic technique, it has to be demonstrated that the registrations obtained with the automatic technique are at least as good as obtained with the manual approach. In that process it is useful to have the reproducibility level of the manual technique available as a benchmark.

The aim of this study was to investigate the reproducibility of the interactive registration technique currently in use for a large radiotherapy pediatric patient population.

## MATERIAL AND METHODS

### Data acquisition

Most of the patients treated for primary brain tumors in St. Jude Children's Research Hospital are imaged using both CT and MR. CT images were acquired using a Siemens Somatom Plus 4 (Siemens Medical Systems, Iselin, NJ). These images were intended for treatment planning and the entire head and neck were imaged. The number of slices varied between 45 and 95 slices, dependent on the size of the patient. Slice thickness and spacing was 3 mm. The image matrix was 512 by 512 and pixel sizes varied between 0.5 and 0.92 mm. Patients were imaged in treatment position and were either sedated or immobilized using a mask or frame.

MR images were acquired on a Siemens Magnetom SP63 or Vision (Siemens Medical Systems, Iselin, NJ), using standard Siemens quadrature head coils. Standard imaging protocols were used to obtain *T*1 [pulse sequence repetition time (TR)=165 ms, echo time (TE)=4.7 ms] or T2(TR=9000 ms, TE=119 ms) weighted images. Subsequently, a 3D reconstruction was calculated. These images were mainly obtained for diagnostic purposes, and after registration were used in treatment planning. Usually the entire head was imaged, but for five patients studied, only part of the brain was scanned. The number of slices varied between 19 and 65. The slice thickness and spacing was 3 mm. The image matrix was 256 by 256. The pixel size was either 0.82 mm square or 0.91 mm by 0.82 mm. During MR image acquisition, the patient was not in treatment position nor wearing any immobilization devices. The very young patients were sedated and every effort was made to reassure and relax the other children. No corrections were applied to the MR images.

The study reported here was performed to ensure the quality of the various ongoing clinical study protocols that have been approved by the institutional review board. Parents or guardians approved usage of clinical data for research purposes prior to treatment. No image selection took place and all images were entered in this prospective trial as they arrived in the clinic. The age of the patients included in this study ranged from 2 years 3 months to 17 years 6 months, with a median age of 10.2 years. The following types of primary tumors were treated: ependymoma (26), medulloblastoma (24), astrocytoma (20), rhabdomyosarcoma (9), and glioblastoma multiforme (2). Twenty‐seven patients had undergone surgical resectioning of the tumor mass, or a biopsy prior to imaging and radiotherapy.

### Registration technique

Image registrations were performed using a separate module of the 3D treatment planning system PLUNC (version 4.0, University of North Carolina, Chapel Hill, NC). This software enabled an operator to apply rigid transformations, translation in the *x* (left‐right), *y* (anterior‐posterior), and *z* (inferior‐superior) direction, and rotations around the *xz, yz*, and *xy* axes, to one of a pair of scanned 3D images. The rotation axis was the center of the image set. Although software like this has been implemented in most commercial treatment planning systems^2^ ^–^ ^4^ and has been developed as a tool by others,^15^ it does vary slightly in the details. In the implementation of the interactive software used here, transverse, sagittal, and coronal views could be put on view in a red and gray color wash display. The operator could move one image, the MR data, by dragging the image over the screen while in one particular view. In order to see all views or to verify the impact of a change at a location not displayed in the current view, the operator had to cycle through the different views. The image could be freely rotated using a dial and in 1 degree increments by clicking in a dial while holding down the shift key.

Before starting the image registration process, the operators changed the window level of the CT images to display only the bony structures. Also the window level of MR images was changed to obtain a good soft tissue contrast, as subjectively judged by the operator.

Although both CT and MR could be displayed using only gray levels, registrations were performed using a color wash display with the MR image in red. Operators preferred using this color wash display because anything in red (soft tissue on MR) could not overlap with anything white (bone on CT). This made it easier to discern any misalignment and immediately drew the attention to important areas. That helped the user to quickly make a mental picture of the 3D movement necessary to reduce the observed misalignment.

### Experiments and data analysis

Over a nine month period, a total of 85 CT/MR brain image registrations were repeated and the time to register the images was recorded. Fifty‐two registrations were repeated at a later date by the same operator. Thirty‐three were repeated by another operator. Three operators were involved in registering images. The first registration was used for clinical purposes. Before repeating the registration, the registration matrix that was, or had been, used clinically, was copied and removed so that the software no longer recognized the existence of the previous registration. The repeated registrations were started from scratch without viewing either the matrix or the registered images prior to the start. The interval between two registrations varied between one week and nine months. This ensured the use of a blinded second observer.

For every registration, the three translation and rotation parameters were obtained, and for every pair the difference between them was calculated. From a translation and rotation difference vector, the difference vector length was obtained separately for both translation and rotation. For all repeats, the rotation difference vector length was plotted as a function of the translation difference vector length and the correlation coefficient was calculated.

The average and standard deviation were calculated for the difference between repeats by the same and a different operator. To test statistical significance of the difference between intra‐ and inter‐operator reproducibility, a two‐sided t‐test was applied to the intra‐ and inter‐operator s.d., for which the standard error of the standard deviation was calculated as $0.71\times s.d./\sqrt{N}$, with *N* as the number of repeats. It was assumed that the reproducibility is fully determined by independent random variations. Then, the 95% confidence interval for registration variation by one observer doing one registration was calculated from the intra‐ and inter‐operator reproducibility, respectively, as twice the standard deviation divided by the square root of the number of repeats and observers.

## RESULTS

**Figure 1:**
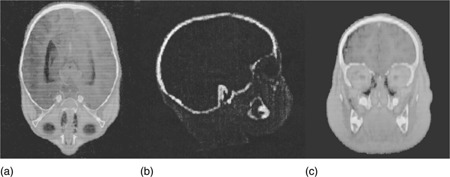
A transverse (a), sagittal (b), and coronal (c) view of a MR data set aligned to a CT set by one of the operators. The displayed bone comes from the CT, the soft tissue from the MR.

Figure 1 shows a transverse (a), sagittal (b), and coronal (c) view of a MR data set aligned to a CT set by one of the operators. The displayed bone comes from the CT and the soft tissue from the MR. The actual image registrations were performed with the MR image presented in a red color wash display.

The standard deviation of the average total translation and rotation was 0.39 mm and 1.7°, and 0.58 mm and 2.8° for inter‐ and intra‐operator repeats, respectively. The maximum difference between registrations was 1.1 mm and 4.1° when repeated by the same operator, and 1.4 mm and 5.8° when repeated by another operator. Image registrations took between 5 minutes and over half an hour for difficult cases, with a mean of 14.3 minutes. Time consuming were the cases where only part of the head, mainly brain, was imaged on MR.

**Figure 2:**
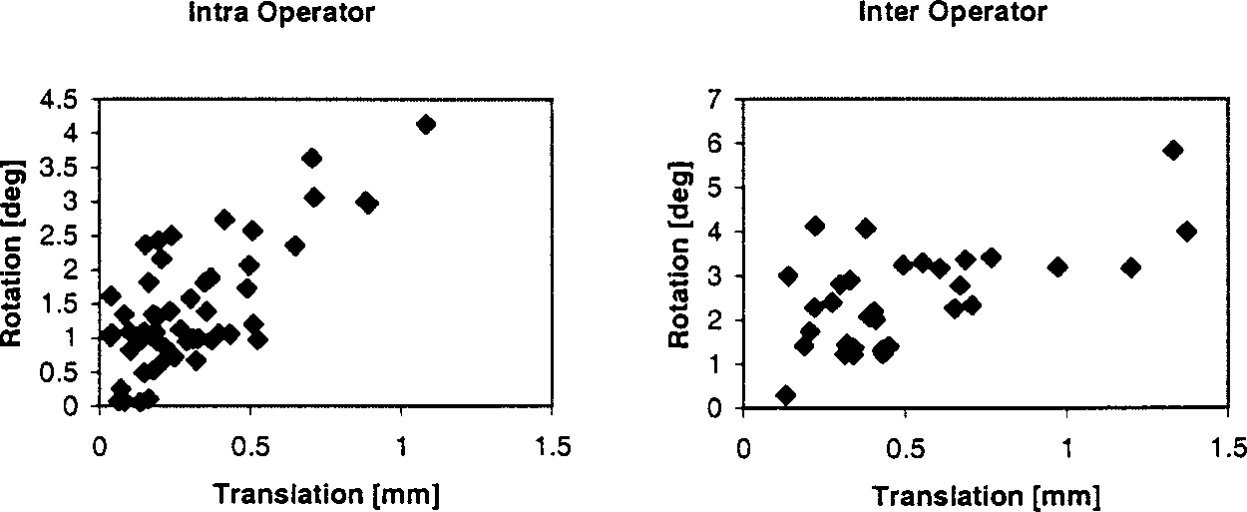
Intra‐ and inter‐operator reproducibility rotation vector magnitude vs. translation vector magnitude for 52 and 33 patients. The correlation coefficient is r=0.73 and r=0.61, respectively.

The magnitude of the difference between two registrations is shown in Fig. 2 for the registration repeated by the same and another operator, for 52 and 33 patients, respectively. In Fig. 2, the magnitude of the rotation difference is plotted vs. the magnitude of the translation. There is a weak correlation between the magnitude of the rotation and translation vector. The correlation coefficients are r=0.73 and r=0.61 for intra‐ and inter‐operator reproducibility, respectively. With standard errors of the standard deviation of 0.037 mm, 0.17°, 0.072 mm, and 0.35°, respectively, the intra‐ and inter‐observer reproducibility is different for the rotation, but not the translation, at the statistical significance level p=0.05.

The variation for the lowest resolution parameters, out of plane translation and rotations, is 2 to 3 times larger than for the in‐plane movements and constitutes, in large part, the total variation (Table I).

**Table I:** Standard deviation for intra‐ and inter‐operator reproducibility using 52 and 33 patients, respectively, of three translations, x, y, and z, and three rotations, around the yz, xz, and xy axes, parameters.

	Intra‐operator [mm]	Inter‐operator [mm]		Intra‐operator [deg]	Inter‐operator [deg]
*x*	0.16	0.26	*yz*	1.24	1.90
*y*	0.11	0.16	*xz*	1.05	1.76
*z*	0.33	0.49	*xy*	0.55	0.95
Total	0.39	0.58	Total	1.72	2.75

## DISCUSSION

Image registration techniques have been around for several years and typically it is stated that images are aligned with 1–2 millimeter accuracy.^6^ ^,^ ^16^ Most studies, however, are based on a small number of patients, on phantoms, or model calculations. This is the first study with a substantial number of patients quantifying the registration reproducibility for CT/MR head images as they are used clinically, and for a technique as it is used clinically. Based on the data in this study, it has to be concluded that an alignment reproducibility, and thus accuracy, of 1–2 millimeter cannot be guaranteed throughout the head volume. With a typical rotation error of 2°, the position error for any point more than 4 cm away from the rotation center exceeds 2 mm.

The registration reproducibility was adequate for the translations. However, the reproducibility of the rotations gave rise to significant displacements, exceeding a few millimeters away from the rotation axis at the center of the image set. This confirmed the subjective impression that, in general, it was quite difficult to decide the exact rotation. Large parts of the skull are smoothly curved and locally almost spherical. Those parts are of limited use for image registration, since it is features like sharp corners and edges on which the registration is based. Additional information might be obtained from using soft tissues in the interactive registration. However, that has the problem that soft tissue contrast in the CT images is rather poor, which is the very reason for using MR images in the first place. As a consequence, the time required to do these image registrations would increase, in particular, because it could become necessary to frequently change the window level of the display. And perhaps more information is utilized in the registration, still that advantage might be more than offset by the increased time consumption in combination with psychological issues like, work pressure, patience, and tiredness.

Similar reasons apply to explain the advantage in using a color wash display. Although the image registration can also be performed displaying both images in gray levels, it would make it harder for the operator to identify misalignments. Since the operators continuously have to cycle through all image slices in transverse, sagittal, and coronal views, this is an important point when taking a technique out of the research setting and using it routinely. In particular, because this interactive technique completely depends on the user, and thus, the above psychological issues. Thus, using some form of color display that is comfortable for the eye will likely result in the most reproducible image alignments in a clinical setting.

This study was based on images as they were used in the clinic. No selection of images was made and for five of the cases, 19 MR slices were acquired which imaged only part of the brain. This significantly reduced the extent of available landmarks on which the registration could be based. No clear impact on reproducibility could be demonstrated, but these registrations took significantly longer than when the entire head was imaged in both sets.

In the literature, it was found that it takes a medically trained operator over half an hour to define up to 8 landmarks in both a CT and MR set.^6^ In that study, the reported accuracy is similar to the reproducibility found in the present study. Hence, interactive registration is quicker than landmark‐based image registration, and the time required to perform an interactive registration in this study is in accordance with the time reported in a recent study for interactive MR‐SPECT registration.^3^

This study demonstrates the effect of image resolution on registration reproducibility. The highest reproducibility is achieved in the directions with the highest image resolution for both translation and rotation. Table I shows that the total reproducibility was predominantly determined by the reproducibility of the low resolution parameters, *z* translation, and *xz* and *yz* rotations. This implies that the registration reproducibility could be improved by decreasing the image slice thickness. The small difference in the magnitude for translation in *x* and *y* direction, and rotation around the *xz* and *yz* axis may be accounted for by the observation that a number of the MR images had pixel sizes 0.91 mm by 0.82 mm.

Intra‐ and inter‐observer data are reported separately. Although the main purpose of the study was to quantitate the reproducibility of the image registration technique in clinical use, because intra‐ and inter‐observer data are potentially different they need to be reported separately. A significant difference would signal that internal discussions regarding registration criteria are required.

The correlation coefficient between the translation and rotation vector magnitude was r=0.73 and r=0.61 for intra‐ and inter‐operator reproducibility. A high correlation coefficient could suggest that the operators chose certain landmarks and compensated a translation error with a second rotation error, while ignoring or forgetting about previous locations and views. If that were the case, a human observer could observe smaller errors than the ones reported in this study. And that would mean that observing the registered image would be a subjective, but nevertheless valid approach, for validating automatic image registration techniques. Unfortunately, in view of the magnitude of the reported error and the weakness of the correlation, it cannot be concluded or ensured that the registration error is 1–2 millimeters by a mere visual check of the registration result of an automatic technique.

In this study, images of pediatric patients were registered. Although continued growth would cause difficulties matching follow up studies acquired after a relatively long time, this is not important in this study given the short interval between acquiring the MR and CT images. However, motion artifacts could potentially be a problem, since for younger patients, it can be expected that lying still during the entire length of a scan is more difficult than for adult patients. It is standard for the very young patients to be sedated before the MR scan, and although upon close inspection for a few patients, motion artifacts could be discerned, the registration reproducibility is mostly determined by the difficulty of aligning a 3D object.

## CONCLUSION

The interactive registration as described is relatively time consuming and 1–2 millimeter reproducibility is not achieved using the approach. The data is a benchmark when introducing automated techniques and underlines the need for doing so. The value of visual inspection of the result of an automatic technique, using a display as described, is limited to detecting outliers. There is a clear image resolution effect on registration reproducibility, suggesting that reducing slice thickness could considerably improve registration reproducibility.

## ACKNOWLEDGMENTS

The author wishes to thank Douglas Coffey and Pradeep Patra for their cooperation, John Conway and David Barber for fruitful discussion, and Weston Park Hospital (Sheffield, UK) for the hospitality during part of this work. This investigation was supported in part by the James Morrison Research grant (Weston Park Hospital, Sheffield, UK), a Whitaker Foundation grant No. R29 CA65606 and Cancer Center Support CORE Grant No. P30 CA 21765 from the National Cancer Institute.
